# Gene Editing Targeting the DUX4 Polyadenylation Signal: A Therapy for FSHD?

**DOI:** 10.3390/jpm11010007

**Published:** 2020-12-23

**Authors:** Romains Joubert, Virginie Mariot, Marine Charpentier, Jean Paul Concordet, Julie Dumonceaux

**Affiliations:** 1 NIHR Biomedical Research Centre, University College London, Great Ormond Street Institute of Child Health and Great Ormond Street Hospital NHS Trust, London WC1N 1EH, UK; romain.joubert@kcl.ac.uk (R.J.); virginie.mariot@ucl.ac.uk (V.M.); 2 Genome Structure and Instability laboratory, CNRS UMR 7196, Inserm U1154, National Museum of Natural History, 75005 Paris, France; marine.charpentier@univ-rennes1.fr (M.C.); jean-paul.concordet@mnhn.fr (J.P.C.); 3 Northern Ireland Center for Stratified Medicine, Biomedical Sciences Research Institute, Londonderry BT47 6SB, UK

**Keywords:** facioscapulohumeral dystrophy, FSHD, TALEN, CRISPR-Cas9, gene editing, muscle, polyadenylation, D4Z4, DUX4

## Abstract

Facioscapulohumeral dystrophy (FSHD, OMIM: 158900, 158901) is the most common dystrophy in adults and so far, there is no treatment. Different loci of the disease have been characterized and they all lead to the aberrant expression of the DUX4 protein, which impairs the function of the muscle, ultimately leading to cell death. Here, we used gene editing to try to permanently shut down DUX4 expression by targeting its poly(A) sequence. We used transcription activator-like effector nucleases (TALEN) and CRISPR-Cas9 nucleases in vitro on FSHD myoblasts. More than 150 TOPO clones were sequenced and only indels were observed in 4%. Importantly, in 2 of them, the DUX4 poly(A) signal was eliminated at the genomic level but DUX4 mRNA was still produced thanks to the use of a non-canonical upstream poly(A) signal sequence. These experiments show that targeting DUX4 PAS at the genomic level might not be an appropriate gene editing strategy for FSHD therapy.

## 1. Introduction

Facioscapulohumeral dystrophy (FSHD, OMIM: 158900, 158901) is one of the most frequent forms of inherited muscular dystrophy with an estimated prevalence of 1:8000 to 1:20,000 [1]. FSHD patients present a selective and asymmetric atrophy of facial, shoulder, arm and leg muscles. The genetic disease is usually transmitted via an autosomal dominant mode of inheritance. The vast majority of patients (95%, FSHD1) show partial deletion of a 3.3 kb tandemly repeated sequence named D4Z4 located on the sub-telomeric region of chromosome 4 [2]. In the general population, the number of repeats varies from 11 to 150, whereas FSHD1 patients carry between 1 and 10 repeats. The 5% remaining patients (FSHD2) do not display this D4Z4 contraction but present mutations in epigenetic modifier genes including SMCHD1, DNMT3B and LRIF1 [3,4,5]. Both FSHD1 and 2 patients are phenotypically indistinguishable and share common features. The mutations cause a chromatin relaxation leading to a D4Z4 derepression associated with aberrant expression of the DUX4 transcription, whose ORF is within each D4Z4 repeat [3,6,7]. When expressed in mucles, DUX4 is highly toxic, leading to a transcriptional deregulation cascade, with ultimately myopathic effects (for review see [8]) and the spreading of the pathological phenotype [9,10]. DUX4 is expressed at very low levels but is robustly found in adult and fetal FSHD biopsies and muscle cells [11,12,13]. There is now a scientific consensus that FSHD is caused by this aberrant expression of DUX4 is muscles. 

At least 5 different *DUX4* mRNAs have been characterized leading to either a truncated protein (DUX4-s) or a full-length protein (DUX4-FL) [11]. The *DUX4-s* isoform is non-pathogenic, rarely found in muscle cell or biopsies and results from the use of a cryptic splicing site located within the ORF, leading to a truncated and non-functional protein. The two *DUX4-FL* isoforms are pathogenic, only differ by the presence or the absence of intron 1 in the 3′UTR of the transcript, and lead to the same toxic protein (for review [14]). All these 5 isoforms use the same PAS located outside of the D4Z4 region, in the subtelomeric region (called PLAM) of the chromosome 4 that is only present in the 4qA variant of chromosome 4, [15,16,17,18]. 

Because we and others have previously shown that the use of antisense oligonucleotides targeting the 3′ end elements involved in *DUX4* mRNA processing is an efficient therapeutic strategy for FSHD [19,20,21,22], in this study, we performed gene editing using transcription activator-like effector nuclease (TALEN) and CRISPR-Cas9 technology to permanently inhibit DUX4 expression by targeting its poly(A) signal sequence (PAS). Even though TALENs initially proved highly efficient nucleases for gene editing, CRISPR-Cas9 has now superseded TALEN technology due to the simplicity of designing guide RNAs for sequence-specific DNA cleavage by Cas9 proteins, the only constraint being the presence of a PAM motif, specific to the different Cas9 proteins available for gene editing, next to the target sequence. Indeed, gene editing has been successful in correcting mutations responsible for genetic diseases in several proof-of-principle studies including for muscular diseases [23].

Here, we designed five TALEN pairs and seven guide RNAs targeting DNA cleavage at the polyadenylation signal of DUX4. FSHD cells were transfected with the nucleases and an oligonucleotide to favour homology directed repairs (HDR). More than 150 clones were generated and sequenced but only two presented an insertion of the oligonucleotides destroying the DUX4 PAS. However, a redirection of the polyadenylation site was observed in the modified clones, similar to what we previously observed with an antisense strategy targeting the DUX4 pre-mRNA with antisense oligonucleotides [19]. Whereas it is possible to permanently destroy the polyadenylation site of DUX4, this may lead to the use of an alternative PAS, and DUX4 may not be efficiently silenced. 

## 2. Results

### 2.1. Nuclease Design and Selection

**Figure 1:**
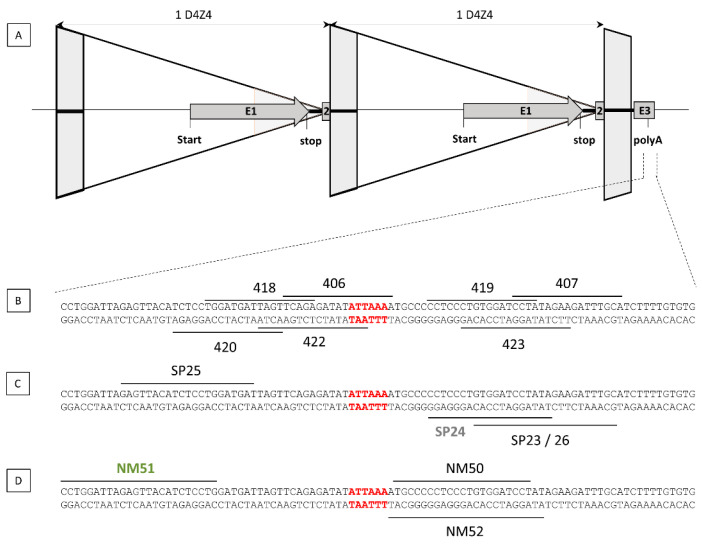
TALEN and gRNA design. TALENs and guide RNAs were designed to target the D4Z4 region (**A**). 5 TALEN pairs were designed, and their positions are indicated (**B**). Six single guide RNAs (sgRNAs), among them, 4 are complexed to *Streptococcus pyogenes* Cas9 endonuclease (**C**) and 2 to *Neisseria meningitides* Cas9 (**D**). The PAS is indicated in red.

To achieve permanent inhibition of DUX4 by targeting the polyadenylation site of DUX4 (Figure 1A), we designed five TALEN pairs (Figure 1B) and six single guide RNAs (sgRNAs), four for *Streptococcus pyogenes* Cas9 endonuclease recognizing the NGG and NAG PAM motifs (Figure 1C) and two for *Neisseria meningitides* Cas9 endonuclease recognizing the NNNNGATT PAM motif (Figure 1D). 

**Figure 2:**
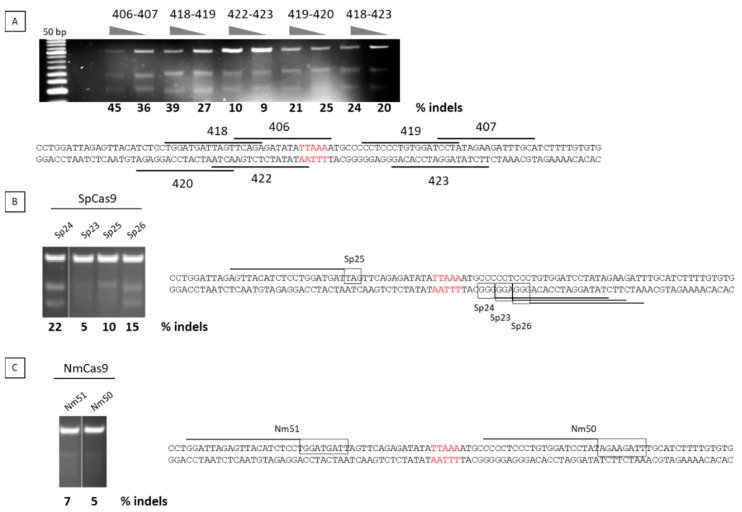
Nuclease selection. U2OS cells were transfected with expression plasmids for TALENs (**A**) or for guide RNAs and cognate *Streptococcus pyogenes* Cas9 (SPCas9 in **B**) or *Neisseria meningitides* Cas9 (NmCas9 in **C**) proteins. Two days after transfection, DNA was extracted and the frequency of indels in the targeted DUX4 PAS was analysed by the T7E1 assay. The target region was amplified by PCR, then PCR products were denatured, followed by re-annealing, leading to a population of double strand fragments, some of which containing mismatches that are detected and cleaved by the T7 endonuclease. The cleavage products are visible on an agarose gel. PCR product is 500 bp long and after T7E1 digestion, expected bands are roughly 200 and 300 bp long depending on the position of mutations induced by the nuclease tested. The proportion of indels was estimated from quantification of the different bands corresponding to non-cleaved and cleaved PCR products.

The efficiency of both TALENs and guide RNAs was tested by transfection of the corresponding plasmids into U20S cells. We have verified that U20S cells contain the 4qA allele and can therefore be used for testing nucleases that target the DUX4 PAS specific to 4qA. We used the T7 endonuclease 1 assay to evaluate the nuclease efficiencies. After transfection of the cells with the nuclease plasmids, cells were harvested in bulk and a PCR flanking the PAS region was realized to determine the percentage of indels using the T7 endonuclease 1 that that specifically cleaves DNA hetero- but not homoduplexes. For the TALENs, the highest percentage of indels was 45% and was achieved with pair 406/407 (Figure 2A), whereas with CRISPR/Cas9, the highest percentage was achieved with SPCas9/sgRNA24 with 22% (Figure 2B,C). The TALEN pair 406/407 was therefore chosen for further experiments.

### 2.2. Transfection of the TALEN Expression Plasmids into FSHD Cells

**Figure 3:**
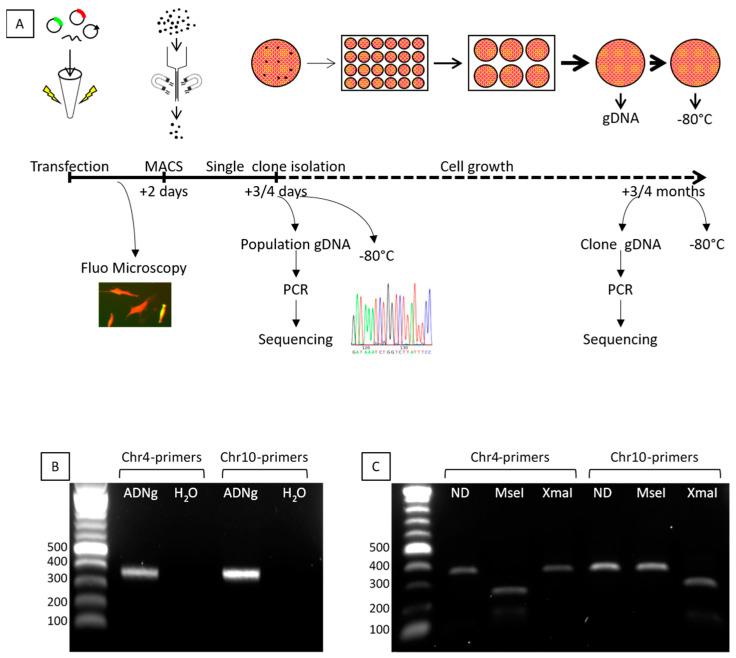
Flow chart and primers validation. The flow chart is described (**A**). Genomic modifications are assessed by sequencing, Primers were designed to specifically recognize chromosomes 4 or 10. The PCR products are similar in length (**B**) but digestion by MseI (present only on chromosome 4) or XmaI (present only on chromosome 10) confirmed the PCR specificity (**C**).

The protocol we followed is described in Figure 3A. Briefly, immortalized FSHD cells were transfected with the TALEN pair 406/407, single-stranded DNA donor oligonucleotides (including a miR1) and a CD4-expressing plasmid for the selection of the transfected cells using magnetic beads coupled to anti-CD4 antibodies (MACS column). After enrichment, 3 to 17% of the transfected cells were harvested and genomic DNA (gDNA) of the population was sequenced after cloning of the PCR products into TOPO-TA plasmids. More than 150 TOPO clones were sequenced and small nucleotide insertions or deletions (indels) were observed in 4% of the TOPO clones. We next performed clonal expansion, and isolated and sequenced 277 cellular clones. Importantly, because chromosomes 4 and 10 share 98% homology, a PCR differentiating these 2 chromosomes was absolutely required. We designed two pairs specifically recognizing each chromosome. Both pairs give rise to PCR products similar in length (Figure 3B). We confirmed the specificity of the PCR products after digestion by MseI and XmaI restriction enzymes (Figure 3C). Indeed, MseI restriction site is present only in chromosome 4, whereas the XmaI site is present only on chromosome 10. PCR products were also sequenced to definitely assess the specificity of the primers.

### 2.3. Selection of the Genetically Modified Clones

**Figure 4:**
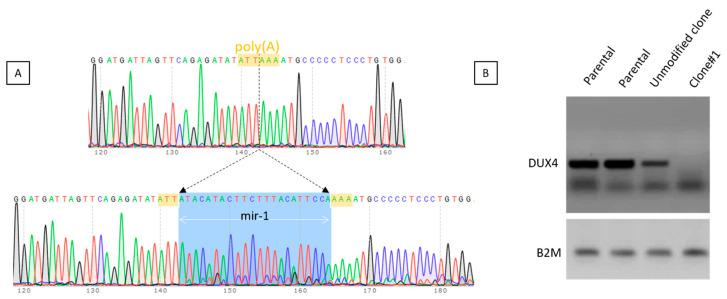
Characteristics of clone#1. This clone was selected after transfection of TALEN pairs 406-407 with a miR-1 ssDNA oligonucleotide. (**A**): The miR-1 sequence (in blue) is inserted within the PAS (in yellow). (**B**): Myoblasts from clone#1 were treated with 0.4 mM chaetocin and harvested 24 h later. DUX4 expression was assessed after RT-PCR Parental FSHD cells and one unmodified clone obtained in parallel to clone#1 were used as controls.

Only two clones showed targeted modifications of the DUX4 PAS. The clone#1 presented the insertion of the mir1 sequence into the DUX4 PAS as a result of DNA repair using ssDNA oligonucleotide miR-1 co-transfected with the TALEN pair 406/407 (Figure 4A). We chose to incorporate the target sequence of miR-1 into the PAS site because this miRNA is specifically expressed during differentiation in parallel to DUX4 mRNA [24] and the addition of target sequences for miR-1 may be sufficient to inhibit the translation of residual DUX4 mRNA.

The capability of the clone to express DUX4 was assessed on myoblasts treated with chaetocin, an inhibitor of the SUV39H1 methyltransferase responsible for establishing the chromatin repressive mark H3K9me3 and leading to a decreased H3K9me3 at D4Z4 and to the expression of *DUX4-FL* [25,26]. We observed that after disruption of the PAS by insertion of the miR-1 sequence, no DUX4 mRNA was detected (Figure 4B). It was not possible to further investigate DUX4 expression in this clone because we noted a decrease in its proliferative capacity and it stopped dividing.

**Figure 5:**
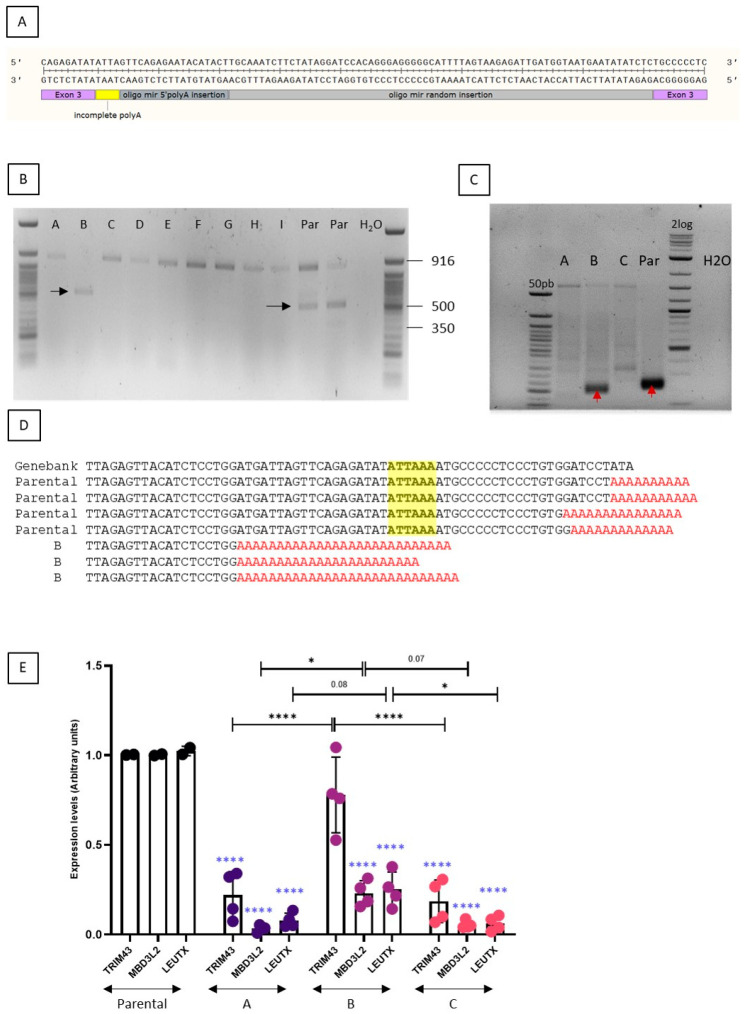
Characteristics of clone#2. (**A**): This clone was selected after transfection of TALEN pair 406-407 with both the miR-5′ and miR-random. The chromosomal modifications are shown. (**B**): DUX4 mRNA expression was assessed by RT-PCR in different wells with cells of the clone carrying the modification and in the parental unmodified FSHD cells (Par). (**C**): 3′RACE nested PCR using forward primers located in Exon 3 shows the redirection of the cleavage site in the modified clone (B) compared to the parental cells (Par), as indicated by the red arrows. (**D**): The sequence shows the redirection of the cleavage site. The PAS is highlighted in yellow. (**E**): Expression levels of 3 genes downstream of DUX4 (TRIM43, MBD3l2 and LEUTX) were measured in parental cells and wells A, B and C. Data are presented as means ± SD; ****: *p* < 0.0001; *: *p* < 0.05 by one-way ANOVA with Tukey’s post hoc test. When the parental cells are compared to the clone, statistical analysis are presented in blue. Other analyses are indicated on the graph. Par: parental.

The second clone presented a complex genotype with a recombination between the ssDNA oligonucleotides miR-random and miR-upstream and the genomic DNA (Figure 5A). 

In order to determine if DUX4 expression was maintained in this clone, we performed a RT-PCR using primers flanking the two introns of DUX4 which can amplify both the DUX4-FL isoforms (DUX4-FL1: 368 bp and DUX4-FL2: 504 bp). In the parental cells, DUX4-FL2 only was amplified (Figure 5B). In the clone, it was not possible to amplify DUX4 mRNA most of the time, but a band corresponding to DUX4-FL2 was also detected (Figure 5B). A redirection of the polyA and/or cleavage site was thus investigated by 3′RACE nested PCR. A switch was observed in the mutated clone (Figure 5C) and the sequence of this lower band revealed that the cleavage occurred ~40 nt upstream of the canonical one (Figure 5D), as we previously observed with the use of some antisense oligonucleotides targeting the polyadenylation site of DUX4 pre-mRNA [19]. This result indicates that while it is technically possible to eliminate the DUX4 poly(A) signal sequence, it does not necessarily result in DUX4 silencing as another alternative poly(A) site may be used. 

We also measured the expression of three well characterized DUX4 downstream genes: TRIM43, MBD3L2 and LEUTX (Figure 5E). In wells A and C, we observed a 95% (*p* < 0.0001) decrease of MBD3L2 and LEUTX, and a 85–90% (*p* < 0.0001) decrease of TRIM43. In well B, expression of these genes was also reduced but to a lower extent, a 78% ± 7 decrease for MBD3L2 (*p* < 0.0001), a 75% ± 9 decrease for LEUTX (*p* < 0.0001) while the apparent decrease was TRIM43 was not statistically significant. When wells A and C are compared to well B, statistical differences were observed for TRIM43 expression (*p* < 0.0001). For MBD3L2 and LEUTX, a 68% decrease or more was observed between well B and wells A/C (Figure 5E). The use of the alternative PAS in well B, leads to a lower activation of the genes downstream of DUX4 when compared to the parental cells but to a higher expression when compared to wells A/C. 

## 3. Discussion

During the past 10 years, several strategies aiming at inhibiting DUX4 expression have been developed (for review see [27]) and the poly(A) signal was already successfully targeted to inhibit DUX4 expression [19,21]. Targeting the key elements of DUX4 mRNA’s 3′UTR is attractive for several reasons: (i) correct polyadenylation of mRNAs is required for their stability, nuclear export and efficient translation, and targeting the polyadenylation signal can result in decreased gene expression (for review see [20]). (ii) DUX4 expression is common in FSHD1 and FSHD2 patients and therefore targeting DUX4 will be beneficial to all FSHD patients. (iii) Contraction of the D4Z4 array on chromosome 10, which carries a single point mutation in the polyadenylation signal sequence found on chromosome 4, does not lead to DUX4 expression [18]. (iv) DUX4 expression has been described in keratinocytes [28] and in EBV-immortalized FSHD lymphoblastoid cell lines [29]. Moreover, DUX4 expression [28,29] is normally suppressed in post-mitotic tissues with the exception of testis where alternative 3′exons are used with a polyadenylation signal in exon 7 that is approximately 6.5 kb further telomeric to the one used in muscles [11]. Moreover, DUX4 might act as a transcription factor to activate cleavage-stage-specific gene expression and may be activated in the primary spermatocytes during spermatogonia and it may therefore be important to target DUX4 in muscles preferentially. Targeting the polyadenylation signal located in exon 3 may lead to the inactivation of the pathogenic DUX4 only, the one that is expressed in the skeletal muscle, whereas the DUX4 mRNA expressed in testis may be spared. 

We decided to not incorporate a resistance gene in our constructs in order to be as close as possible to the context of a clinical application. After transfection, we detected two clones with genetic modifications. This might be due the absence of selection and also to the difficulty to transfect myoblasts. In order to increase our chance to inhibit DUX4 expression, we co-transfected a double strand oligonucleotide carrying the miR1 target sequence: in the case of the use of an alternative polyadenylation (APA) signal located downstream of the regular one, DUX4 translation would have been strongly impaired by its presence in the DUX4 mRNA. Indeed, miR1 is a well described myomir, which promotes myoblast-to-myotube differentiation and is up-regulated during this process [30]. 

It was technically possible to destroy the DUX4 PAS at genomic level, but unfortunately, a redirection of the polyadenylation site, leading to a full length DUX4 mRNA was observed, leading to a lower activation of several genes downstream DUX4. In the literature, only two DUX4 APA sites have been described: one in exon 7 [11], which may be used in testis only and one in exon 3, located ~40 nt upstream of the canonical one, and sometimes observed after treatment of FSHD cells with antisense oligonucelotides targeting the DUX4 PAS [19]. Interestingly, here we report the same polyadenylation site redirection as with the antisense oligonucleotides, suggesting that a steric congestion at the polyadenylation signal and cleavage site provided by the antisense oligonucleotide may not be responsible for the redirection. This rather suggests the presence of an unknown, non-canonical poly(A) signal upstream of the ATTAAA, which may be used in the absence of the canonical poly(A) signal sequence. The use of this proximal APA has been described for other mRNAs in the literature (For review see [20]). It leads to a shorter mRNA but to the same protein since exon 3 is a non-coding exon. Our results suggest that targeting the DUX4 poly(A) signal using TALENs to destroy the PAS is possible but may not lead to an efficient extinction of DUX4 and may not be a suitable strategy for FSHD. A strategy targeting both the canonical and the alternative PAS detected here could give a more robust extinction of DUX4 expression. This would be possible by inducing the corresponding deletion with programmable nucleases, for example using two guide RNAs flanking the PAS and APA sequences with Cas9.

## 4. Material and Methods

### 4.1. TALEN and Guide RNA Plasmid Design and Validation

TALEN and guide RNA expression plasmids were constructed using standard molecular biology cloning methods as detailed in [31]. Expression plasmids were transfected into U2OS (established in 1967 from a moderately differentiated sarcoma of the tibia of a 15-year-old girl [32,33]) cells using Amaxa-mediated electroporation (Lonza, Colmar, France) and DNA cleavage activity was assayed indirectly by the T7E1 assay. Primers were hFSHDcr12 fw (5′ gtctgtctttgcccgcttcc 3′) and hFSHDcr12 rev (5′ tgcctacactctgcctacagga 3′). PCR product is 500 bp long and after T7E1 digestion, expected bands are roughly 200 and 300 bp long depending on the position of mutations induced by the nucleases tested.

### 4.2. Cell Culture

The immortalized myoblasts (54-2) were grown as previously described (Krom et al. 2012). Briefly, cells were grown in 64% DMEM, 16% 199 medium, 20% FBS, supplemented with fetuin 25 µg/mL, β-FGF 0.5 ng/mL, hEGF 5 ng/mL, Insulin 5 µg/mL, Dexametasone 0.2 µg/mL, Gentamycin 50 µg/mL. The transfection was performed using Neon Transfection System (Life technologies, Courtaboeuf, France) on 10^6^ cells with 0.21 µg of both TALEN plasmids, 0.2 µg of CD4 expression plasmid, and 0.38 µg of single-stranded donor oligonucleotide (oligonucleotide miR-1 5′GATTAGAGTTACATCTCCTGGATGATTAGTTCAGAGATATATTatacatacttctttacattccaAAAATGCCCCCTCCCTGTGGATCCTATAGAAGATTTGCATCTTTTGTGTG3′; oligonucleotide miR-random 5′GATTAGAGTTACATCTCCTGGATGATTAGTTCAGAGATATATTcattaccatcaatctcttactAAAATGCCCCCTCCCTGTGGATCCTATAGAAGATTTGCATCTTTTGTGTG3′; oligonucleotide miR-1-upstream 5′ACCTGGATTAGAGTTACATCTCCTGGATGATTAGTTCAGAGAatacatacttctttacattccaTATATTAAAATGCCCCCTCCCTGTGGATCCTATAGAAGATTTGCATCTTTT3′). After 48 h of incubation, cells were enriched on MACS columns with a CD4 antibody (Miltenyi Biotec, Paris, France) following manufacturer’s recommendation. Monoclonal cell population was performed by limiting dilutions, 3 or 4 days later according to the confluency. Briefly, cells were diluted to seed around 1 cells/well in 96-well plate. After checking under microscope, and scanning the entire plate, wells with only one cell colony were selected and, generally after 1–2 weeks, expanded when confluent in 24-well plate, 6-well plate, and 10 cm dish. For chaetocin treatment, when myoblasts reaching 80% confluency were treated with 0.4 mM chaetocin (Sigma-Aldrich C9492, Saint Quentin Fallavier, France). 

### 4.3. Genomic DNA Analysis

Genomic DNA was extracted from myoblast culture using EZNA tissues DNA kit (VWR) according to the manufacturer’s instruction. PCR to amplify DUX4 genomic sequence were performed using GoTaq Green Master Mix (Promega, Madison, WI, USA) and primers specific of chromosome 4 (5′-AGCTGCCAGCGCGGAGCT-3′ and 5′-TGATCACCGAAGTTATGTAAAC-3′), or chromosome 10 (5′-AGGTGCCAGCACGGAGCG-3′ and 5′-TGATCACCGAAGTTATGTAAAT-3′). The size of the resulted amplicon is 362bp and after digestion by MseI and XmaI, the chromosomic origin can be identified because MseI is specific of chromosome 4 and XmaI is specific of chromosome 10. The different amplicons were eventually cloned using Topo-TA cloning kit (Life technologies) and sequenced with M13 primer (5′-GTAAAACGACGGCCAG-3′).

### 4.4. RT-PCR and RT-qPCR

RT-PCR has been described previously [10,13]. 3′RACE PCR was published in [19]. The primers used for the expression levels of the genes downstream of DUX4 were: B2M (Fwd 5′-CTCTCTTTCTGGCCTGGAGG-3′; Rev 5′-TGCTGGATGACGTGAGTAAACC-3′; amplicon size: 67 bp), TRIM43 (Fwd 5′-ACCCATCACTGGACTGGTGT-3′; Rev 5′-CACATCCTCAAAGAGCCTGA-3′; amplicon size: 100 bp), MBD3L2 (Fwd 5′-CGTTCACCTCTTTTCCAAGC-3′; Rev 5′-AGTCTCATGGGGAGAGCAGA-3′; amplicon size: 142 bp) and LEUTX (Fwd 5′-TGGCTACAATGGGGAAACTG-3′; Rev 5′-CTGCTGCCTCTTCCATTTG-3′; amplicon size: 98 bp). 

### 4.5. Statistical Analysis

GraphPad Prism software was used for statistical analyses. Differences between groups were evaluated by one-analysis of variance (ANOVA) followed by Tukey’s post hoc tests. ****: *p* < 0.0001; ***: *p* < 0.001; **: *p* < 0.01; *: *p* < 0.05.
